# Clinic study on macular epiretinal membrane in patients under the age of 40 years

**DOI:** 10.1186/s12886-023-02813-8

**Published:** 2023-02-25

**Authors:** Nan Wang, Aohua Peng, Shengguo Li, Chun Ding

**Affiliations:** 1 Department of Ophthalmology, the Second Xiangya Hospital of Central South University, 139 Renmin Middle Road, Changsha, 410011 China; 2 Department of Ophthalmology, Changsha Aier Eye Hospital, Changsha, China

**Keywords:** Macular epiretinal membrane, Posterior vitreous detachment, Inner segment/outer segment, Visual acuity, Central foveal thickness, Intravitreal triamcinolone acetonide

## Abstract

**Background:**

To describe the risk factors and clinical characteristics of macular epiretinal membrane (MEM) disease in patients up to the age of 40 years and to evaluate the therapeutic effect of IVTA on MEM.

**Methods:**

Clinical records were reviewed and the etiology of each case and the age distribution data were collected in this retrospective, cohort study. The clinical characteristics of MEM and the factors affecting VA were analyzed. Additionally, we contrasted the effects of MEM peeling with and without intravitreal triamcinolone acetonide on visual acuity (VA) and central foveal thickness (CFT).

**Results:**

In young patients, the incidence of partial posterior vitreous detachment (P-PVD) was considerably higher in IMEM than SMEM (*P* = 0.007). Furthermore, patients with stage 3 MEM had lower BCVA values than patients with stage 4 MEM (*P* < 0.001). Patients who live in urban had lower BCVA values than patients in rural (*P* < 0.001). Patients with IS/OS integrity had lower BCVA values than patients without IS/OS integrity (*P* < 0.001). The BCVA values in patients with IMEM were significantly lower than those of patients with SMEM (*P* < 0.001). BCVA was associated most commonly with etiology (*P* = 0.001), followed by region (*P* = 0.002). All patients had a decrease in logMAR Vas and CFT, but the combination of intraoperative IVTA resulted in a more significant decrease in logMAR Vas (*P* = 0.007) and CFT (*P* = 0.046).

**Conclusion:**

In young patients, the incidence of P-PVD was significantly higher in IMEM cases than in SMEM cases. The region, MEM stage, IS/OS integrity, and etiology influenced VA. Etiology was associated most commonly with BCVA. In individuals under 40, the combination of intraoperative IVTA resulted in a more significant decrease in logMAR Vas and CFT.

## Background

Macular epiretinal membrane (MEM) disease is a fibrocellular proliferation on the surface of the inner limiting membrane (ILM) that causes structural changes in the macula, resulting in metamorphopsia and vision loss. Clinical manifestations vary from asymptomatic cellophane membranes to fibrous, contractile membranes associated with blurred vision, monocular diplopia, metamorphopsia, micropsia, decreased visual acuity (VA), and loss of central vision [1–3].

The exact mechanism of MEM remains to be determined. One hypothesis was that separation of the vitreous membrane from the retina, or vitreoretinal dissection, results in inflammation-mediated proliferation of glial cells, fibrous astrocytes, fibroblasts, myofibroblasts, and macrophages on the surface of the retina. The incidence of MEM was reported as 1.1% per year, and the estimated prevalence was as high as 28.9% [4–18]. MEM is usually classified as idiopathic (IMEM) or secondary (SMEM). MEM most commonly occur in patients older than 50 years.

Because of the lower incidence of MEM disease in young patients, most of the current research on MEM has focused on people over 40 years of age. MEM can also occur in young patients after trauma or eye diseases such as uveitis, retinal vascular disease, or tumors. There had some studies of MEM in patients up to 40 years old [19–23]. However, current research on the risk factors and pathogenesis of MEM in young patients is limited, such as whether posterior vitreous detachment (PVD) in young patients with IMEM plays a key role in the formation of MEM. The risk factors for VA in young patients with MEM had not been reported, and the key factors influencing the patients' VA were also seldom reported. It is very important to clarify the pathogenesis of MEM in young patients to determine the appropriate follow-up treatment, since currently there is no unified standard.

MEM is often complicated by macular edema, which can be removed by pars plana vitrectomy and membrane peeling. Still, macular edema is more difficult to resolve, so in the perioperative management of adult patients, some clinicians use triamcinolone acetonide (TA) during surgery to help restore macular morphology and prevent further progression of macular edema. But the intraoperative use of TA in young MEM patients were less reported [24–28].

Therefore, the purpose of our study was to describe the risk factors and clinical characteristics of MEM in patients up to the age of 40. And we compared the effect of intravitreal triamcinolone acetonide (IVTA) on central foveal thickness (CFT) and VA in MEM patients.

## Methods

This was a retrospective study conducted in accordance with the Declaration of Helsinki and approved by the Ethics Committee of the Second Xiangya Hospital. We reviewed the medical records of all patients under 40 years of age diagnosed with MEM at the Second Xiangya Hospital from January 1, 2011 to July 31, 2021. The demographic characteristics of participants such as age, gender, place of residence, and systemic comorbidities were collected.

Ophthalmic records were carefully reviewed for VA, ocular misalignment, and anterior segment and fundus findings. Fundus photography, B-scan ultrasonography, and optical coherence tomography (OCT) were also reviewed. We used OCT images to examine the extent of MEM, and fundus photography to evaluate the turbidity of MEM, and analyzed the clinical characteristics of MEM and the factors affecting VA. Each case was classified as IMEM or SMEM, and the age distribution and etiology of each case analyzed. Adolescents and children were defined as younger than 19 years old [21] following the epidemiological investigation of MEM by Khaja et al.

In our study, patients under 40 years old were divided into two age groups: younger than 19 years and 19–40 years old. Each age group was subdivided based on MEM etiology into IMEM and SMEM subgroups. The incidence of PVD in patients in the IMEM and SMEM subgroups of the different age groups was calculated. The diagnosis of PVD in MEM cases was based principally on slit-lamp biomicroscopy, OCT, and B-scan ultrasonography. PVD was divided into three categories as follows: no PVD (no-PVD), partial PVD (P-PVD), and complete PVD (C-PVD).

**Fig. 1:**
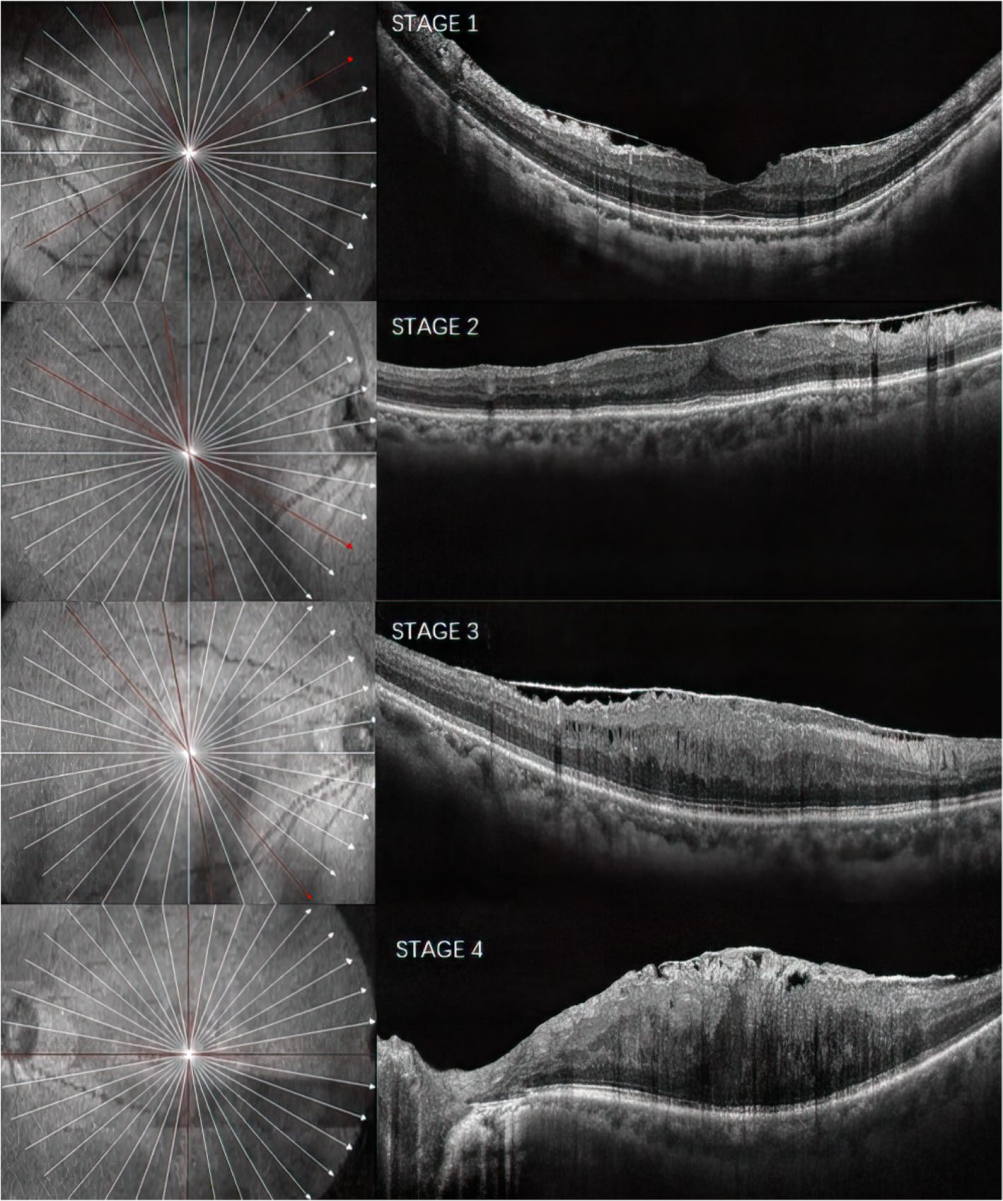
Staging scheme for optical coherence tomography of the MEM. MEM, macular epiretinal membrane

We analyzed the influence of age, gender, eye side, region, MEM Classification stages, IS/OS integrity, MEM type, and etiology on the best-corrected visual acuity (BCVA) of MEM. Optical coherence tomography staging scheme as follows: stage 1, presence of the foveal pit, well-defined retinal layers; stage 2, absence of the foveal pit, well-defined retinal layers; stage 3, absence of the foveal pit, well-defined retinal layers, presence of ectopic inner foveal layers; stage 4, absence of the foveal pit, disrupted retinal layers, presence of ectopic inner foveal layers [29](Fig. 1). We recorded the MEM stage of all MEMs in this study, and used OCT to detect the IS/OS integrity. According to the OCT results, the continuous integrity of IS/OS in the macular fovea without interruption was regarded as complete IS/OS; otherwise, it was regarded as incomplete IS/OS. MEM was divided into two phases according to the fundus photography: the lighter phase, called cellophane macular reflex (CMR), with flashing, watery filaments, and moving light reflections without visible retinal folds, while the more severe form, called preretinal macular fibrosis (PMF), as defined by opaque retinal folds on the inner surface of the retina that appear gray [5].

Three experienced ophthalmologists had done the operations, and whether the operations were combined intraoperatively with IVTA mainly depended on the etiology, vitreoretinal inflammation, and macular edema severity. BCVA, introcular pressure (IOP), slit lamp, and indirect ophthalmoscopy, OCT evaluations were performed during the first visit before the operation and the last visit after the operation. The changes of CFT and BCVA from pre procedure to post procedure with IVTA vs changes from pre procedure to post procedure without IVTA were compared. Patients were adviced to follow-up every 3 months in the first year, every 6 months in the second year, and annually thereafter.

The SPSS 27.0 software was used for statistical analysis. All data were expressed as mean ± standard deviation. Snellen charts were used to measure the BCVA. The best corrected visual acuity (BCVA) was converted to logMAR for statistical evaluation. A lower logMAR vision was a better Snellen equivalent. The logMAR VA values were compared with ANOVA. Independent t test was used to determine the influence of age, gender, eye side, region, PVD, MEM stage, IS/OS integrity, MEM type, and etiology on the BCVA. Multivariable regression (α in = 0.05, α out = 0.10) was performed to identify the most critical factors for BCVA. An independent t-test was used to compare the effect of IVTA on CFT and VA. For all tests, a *P* value less than 0.05 was considered significant.

## Results

In our study, one hundred eyes in one hundred MEM patients less than 40 years old were included, among these, 63 were male. Ninety-four patients had undergone vitrectomy and ILM peeling. IVTA was administered at the end of surgery in 46 patients. The mean BCVA was 1.29 ± 0.777 logMAR. Table 1 shows the clinical attributes of patients with MEM. There were 74 (74%) urban patients and 26 (26%) rural patients. Eleven patients had diabetes, six had hypertension, and one had a cardio-cerebrovascular disease.

**Table 1:** Demographic characteristics of participants according to the presence of MEM, and the result of age and sex adjusted univariate analysis

Variable	No.(%) of eyes
Proportion of age group	
< 19	21
19 ~ 40	79
Proportion of sex distribution	
Male	63
Female	37
Region	
Urban	74
Rural	26
Systemic comorbidities suffered	
Hypertension (No.(%))	6
Diabetes (No.(%))	11
Cardio-cerebrovascular diseases(No.(%))	1

*MEM* Macular epiretinal membrane

The etiology of MEM identified in this study can be seen in Table 2, which showed that 20% of all MEM were in the IMEM subgroup, while 80% were in the SMEM subgroup, with trauma being the most common (23%). The incidence of MEM increased with age, with fewer incidences in patients younger than 18 years (21%) as compared to patients aged 19–40 (79%).

**Table 2:** Etiology of MEM

Etiology	< 19	19 ~ 40	Total
Idiopathic	4	16	20
Coat’s	1	1	2
Eales’	0	6	6
Endophthalmitis	0	1	1
FEVR	0	1	1
Glaucoma	1	0	1
High myopia	0	5	5
Hole	1	9	10
Choroiditis	0	1	1
PDR	0	7	7
Pars plana vitrectomy	3	9	12
Retinal vasculitis	1	3	4
Scleral buckling	0	4	4
Traumatic	8	15	23
Tumor	1	1	2
Unknown	1	0	1
Total	21	79	100

*FEVR* Familial exudative vitreoretinopathy, *PDR* Proliferative diabetic retinopathy, *MEM* Macular epiretinal membrane

We looked at the incidence of PVD categories for IMEM and SMEM cases in each age group separately. As shown in Table 3, there were no C-PVDs in either the IMEM or SMEM subgroups. In the younger than 19 years old group, one eye with No-PVD and three eyes with P-PVD were found in the IMEM subgroup, while 13 eyes with No-PVD and four eyes with P-PVD were found in the SMEM subgroup. In the 19–40 years old group, one eye with No-PVD and 15 eyes with P-PVD were found in the IMEM subgroup, while 21 eyes with No-PVD and 42 eyes with P-PVD were found in the SMEM subgroup. The incidence of P-PVD in the IMEM subgroup was significantly higher than the SMEM subgroup among young patients (*P* = 0.007).

**Table 3:** Comparison of the distribution of the stage of PVD in two age groups in idiopathic MEM and secondary MEM

Etiology	≤ 18	≤ 40	Total
Idiopathic			
No-PVD	1	1	2
P-PVD	3	15	18
C-PVD	0	0	0
Secondary			
No-PVD	13	21	34
P-PVD	4	42	46
C-PVD	0	0	0

*No-PVD* No posterior vitreous detachment, *P-PVD* Partial posterior vitreous detachment, *C-PVD* Complete posterior vitreous detachment, *MEM* Macular epiretinal membrane

We analyzed the influences of age, gender, eye side, region, PVD, MEM stage, IS/OS integrity, MEM type, and etiology on the BCVA in Table 4. The patients with stage 3 MEM had lower BCVA values than patients with stage 4 MEM (0.965 ± 0.716 logMAR vs 1.51 ± 0.744 logMAR, *P* < 0.001). Patients in urban had lower BCVA values than patients in rural (1.077 ± 0.744 logMAR vs 1.888 ± 0.517 logMAR, *P* < 0.001). Patients with IS/OS integrity had lower BCVA values than patients without IS/OS integrity (0.708 ± 0.709 vs 1.486 ± 0.7 logMAR, respectively, *P* < 0.001). The BCVA values in patients with IMEM were significantly lower than those of patients with SMEM (0.535 ± 0.461 vs 1.481 ± 0.724 logMAR, respectively, *P* < 0.001).

**Table 4:** The influence of age, gender, eye side, region, PVD, MEM stage, IS/OS integrity, MEM type, and etiology on the BCVA of MEM

Variables	BCVA (LogMAR)	F	*P*-value
Age		0.046	0.763
< 19y	1.34 ± 0.839		
19 ~ 40y	1.277 ± 0.766		
Gender		0.324	0.555
Male	1.326 ± 0.767		
Female	1.23 ± 0.801		
Eye		0.825	0.722
Right eye	1.263 ± 0.712		
Left eye	1.319 ± 0.848		
Region		4.873	< 0.001
Urban	1.077 ± 0.744		
Rural	1.888 ± 0.517		
PVD		2.713	0.149
No-PVD	1.443 ± 0.916		
P-PVD	1.206 ± 0.684		
MEM stage		0.201	< 0.001
Stage 3	0.965 ± 0.716		
Stage 4	1.51 ± 0.744		
IS/OS integrity		0.007	< 0.001
Yes	0.708 ± 0.709		
No	1.486 ± 0.7		
MEM type		0.561	0.131
CMR	1.055 ± 0.647		
PMF	1.349 ± 0.8		
Etiology		4.802	< 0.001
Idiopathic	0.535 ± 0.461		
Secondary	1.481 ± 0.724		

*PVD* Posterior vitreous detachment, *No-PVD* No posterior vitreous detachment, *P-PVD* Partial posterior vitreous detachment, *MEM* Macular epiretinal membrane, *IS/OS* Inner segment/outer segment, *CMR* Cellophane macular reflex, *PMF* Preretinal macular fibrosis, *BCVA* Best corrected visual acuity

The multivariable regression was performed to determine the factors associated most with the BCVA (Table 5). BCVA was associated most commonly with etiology (*P* = 0.001), followed by region (*P* = 0.002).

**Table 5:** multivariate regression was performed to identify the most critical factors for BCVA

Factors	Beta (95% CI)	*P*-value
Age, < 19 vs 19 ~ 40 years old	-0.021(-0.378, 0.298)	0.816
Sex, male vs female	-0.093(-0.418, 0.120)	0.274
Eye, right eye vs left eye	0.036(-0.202, 0.314)	0.668
Region, urban vs rural	0.309(0.205, 0.881)	0.002
PVD, No-PVD vs P-PVD	0.023(-0.266, 0.341)	0.805
MEM stage, Stage3 vs Stage 4	-0.034(-0.456, 0.348)	0.791
IS/OS integrity, complete vs incomplete	0.223(-0.015, 0.808)	0.059
MEM type, CMR vs PMF	0.002(-0.367, 0.376)	0.981
Etiology, idiopathic MEM vs secondary MEM	0.329(0.261, 1.006)	0.001

*PVD* Posterior vitreous detachment, *No-PVD* No posterior vitreous detachment, *P-PVD* Partial posterior vitreous detachment, *MEM* Macular epiretinal membrane, *IS/OS* Inner segment/outer segment, *CMR* Cellophane macular reflex, *PMF* Preretinal macular fibrosis, *BCVA* Best corrected visual acuity

All patients had a decrease in logMAR Vas and CFT, but the combination of intraoperative IVTA resulted in a more significant decrease in logMAR Vas and CFT (Table 6). The mean logMAR Vas decrease (0.53 ± 0.485 vs 0.296 ± 0.322 logMAR, *P* = 0.007) and CFT reduction (120.87 ± 56.719 µm vs 98.479 ± 50.682 µm, *P* = 0.046) in eyes treated with ILM peeling and IVTA were larger than eyes treatment with ILM peeling. There was no significant IOP elevation in eyes treated with ILM peeling and IVTA (0.674 ± 2.261 mmHg vs 0.333 ± 2.66 mmHg, *P* = 0.506).

**Table 6:** Comparison of VA improvement, CFT reduction, IOP elevation, and followed up between peeling group and peeling with IVTA group

	Peeling	Peeling + IVTA	F	P
BCVA (LogMAR)	0.296 ± 0.322	0.53 ± 0.485	5.000	0.007
CFT (µm)	98.479 ± 50.682	120.87 ± 56.719	1.383	0.046
IOP (mmHg)	0.333 ± 2.66	0.674 ± 2.261	2.963	0.506
Follow up (month)	17.438 ± 11.168	16.391 ± 9.5	1.065	0.627

*VA* Visual acuity, *BCVA* Best-corrected visual acuity, *CFT* Central foveal thickness, *IOP* Introcular pressure, *IVTA* Intravitreal triamcinolone acetonide

## Discussion

This study summarized the clinical features of MEM in young patients under 40 years old. In our cohort, only 21% of patients with MEM were under 19 years old, and the incidence of MEM increased significantly with age. There were more male patients in our study, reflecting the possibility that males were more likely than females to exhibit risky behaviors that increased the opportunities for trauma and subsequent MEM formation. The incidence of IMEM was significantly lower than SMEM in young patients, which was consistent with previous reports that show IMEM was the most common type of MEM in patients over 50 years old, while SMEM was the most common type of MEM in young patients [21].

The current generally accepted mechanism for IMEM begins with a PVD, which causes a mechanical stretch of the retina and can stimulate the production of various types of growth factors. Residual vitreous cortex cells attached to the surface of the ILM are activated by various cytokines, which leads to cell proliferation and the differentiation of myofibroblasts, thus forming epiretinal membrane tissue [30–33]. However, the mechanism of IMEM formation in children and adolescents is still uncertain. Among the reported MEM cases in adolescents and children, the proportion of PVD ranged from 0 to 100% [20, 34–36].

At present, to our knowledge, little research has focused on the incidence of PVD separately in IMEM and SMEM groups in younger patients or quantified the proportion of PVD in the patients with MEM. In our research, we found few cases of C-PVD in patients with IMEM and SMEM, while we did find some patients with No-PVD, and some with P-PVD. We concluded that the possible explanation for these results was that the study patients were young, and the probability of vitreous liquefaction was rare; therefore, the probability of spontaneous C-PVD was zero in our study. However, the incidence of P-PVD in young patients with IMEM was significantly higher than in those with SMEM. We inferred that P-PVD could lead to mechanical stimulation of the retina and destruction of the blood-eye barrier, ultimately leading to the formation of IMEM, which might be similar to the mechanism of IMEM in people over 50 years old. For SMEM patients, the pathogenesis might be related to the breakdown of the blood-retinal barrier and the release of cytokines and growth factors that activate myofibroblasts, a key step in SMEM formation.

In this study, the influences of age, gender, eye side, region, PVD, MEM stage, IS/OS integrity, MEM type, and etiology on the BCVA were also analyzed. Our results showed that patients in urban had better VA than in rural. In our study, most young MEM patients were urban residents, suggesting that economic conditions play a certain role. Urban residents might have greater access to specialist ophthalmologists, and therefore the diagnosis rate of MEM was consequently higher. Most young patients in rural areas often showed very poor vision when visiting hospitals, and most of them had not sought prompt medical treatment, which was associated with the relatively backward economic and medical level in rural areas.

Our research revealed that the BCVA of patients with stage 3 MEM was significantly lower than that of patients with stage 4 MEM. Patients with stage 4 MEM had more obvious thickening of the MEM and stronger tangential traction, which could cause disrupted retinal layers. This could cause retinal edema, bleeding, exudation, and more serious damage to the photoreceptor cell layer, leading to degraded vision. Moreover, our study found that the integrity of the IS/OS layer was closely related to VA. We examined the VA of all young patients and found that the VA of patients with a continuous IS/OS layer was significantly higher than that of patients with a discontinuous IS/OS layer, which was consistent with the results of the previous studies [37]. This finding supports the idea that IS/OS integrity was a significant indicator of visual prognosis.

We also found that etiology was the most critical factor for VA. According to our study, the primary etiology of SMEM in young adults was diverse. In addition to a series of common diseases secondary to trauma, such as retinal holes and post-PPV, rare etiologies including Coats disease, Eales disease, endophthalmitis, familial exudative vitreoretinopathy, high myopia, glaucoma, proliferative diabetic retinopathy, choroiditis, retinal vasculitis, scleral buckling, and tumors. The mean visual acuity was very poor in our study, and the reason for the low visual acuity was that 80% of the patients in this study were SMEM. The SMEM was often combined with other causes affecting vision, and the severity of the primary disease directly affected vision.

Our study revealed for the first time that IVTA (1 mg) improved VA and CFT in patients under the age of 40, as other studies focused on adults over the age of 50. A retrospective study by Konstantinidis et al. demonstrated VA and macular improvement as early as 1-week postoperatively [38], and the study by Angermann et al. suggested that IVTA (1 mg) could accelerate the improvement of VA and CFT [39]. While other studies suggested somewhat different conclusions: patients undergoing IVTA could not significantly improve VA and CFT [40–43]. But our study showed that IVTA could improve the VA and CFT of patients. The possible reasons we hypothesized were: Firstly, some studies showed that the increase in VA was usually from nine months to more than one year after a procedure, previous similar studies focused on six months afterwards [44]. Most of the patients in our study were followed for at least one year. In addition, preoperative VA was also a factor in determining postoperative VA, if it was poor before operation, postoperative visual improvement was also poor [40]. If a MEM patient with good initial vision, the MEM was the most dominant cause of poor vision, MEM removal could improve vision effectively [45]. A recently published study shows no benefits of IVTA [43]. However, that study was a prospective study that only included patients with IMEM, while our study included patients with IMEM (20%) and SMEM (80%), and the patients with SMEM were mostly accompanied by inflammation, so IVTA contributed to the patients' decreased CFT and improved VA. And operations were performed by three ophthalmology specialists with more than twenty years of experience in vitrectomy surgery, who mainly selected whether IVTA was performed or not based on the primary etiology, the degree of vitreoretinal inflammation, and the severity of macular edema. The experienced judgment of the operating surgeon was also an important reason IVTA was effective.

This study has several limitations. Retrospective studies will inevitably face additional surgeon-induced bias, as surgeons' surgical skills and artificial selection of IVTA contribute to changes in postoperative VA and CFT. Due to the retrospective nature of this study, the patients were not followed up regularly. In addition, the sample size was relatively small and the data for each group was unbalanced, which could have led to deviations in the results. Further studies might expand the sample size and optimize the grouping, further refining the age group.

## Conclusion

In a nutshell, our study summarized the clinical features of MEM in young patients under 40 years old. The incidence of IMEM was significantly lower than that of SMEM in young patients. In young patients, the incidence of P-PVD was significantly higher in cases of IMEM than SMEM. The region, MEM stage, IS/OS integrity, and etiology influenced VA. Etiology was associated most commonly with BCVA. The combination of intraoperative IVTA resulted in a more significant decrease in logMAR Vas and CFT.

## Data Availability

The datasets used and analyzed during the current study are available from the corresponding author on reasonable request.

## Abbreviations

MEM: Macular epiretinal membrane
VA: Visual acuity
ILM: Inner limiting membrane
IMEM: Idiopathic macular epiretinal membrane
SMEM: Secondary macular epiretinal membrane
TA: Triamcinolone acetonide
IVTA: Intravitreal triamcinolone acetonide
CFT: Central foveal thickness
OCT: Optical coherence tomography
PVD: Posterior vitreous detachment
P-PVD: Partial posterior vitreous detachment
C-PVD: Complete posterior vitreous detachment
IS/OS: Inner segment/outer segment
BCVA: Best-corrected visual acuity
CMR: Cellophane macular reflex
PMF: Preretinal macular fibrosis
IOP: Introcular pressure
